# *spict*, a cyst cell-specific gene, regulates starvation-induced spermatogonial cell death in the *Drosophila* testis

**DOI:** 10.1038/srep40245

**Published:** 2017-01-10

**Authors:** Ason C.-Y. Chiang, Heiko Yang, Yukiko M. Yamashita

**Affiliations:** 1 Department of Cell and Developmental Biology, Medical School, University of Michigan Ann Arbor, MI 48109; 2 Life Sciences Institute, University of Michigan Ann Arbor, MI 48109; 3 Medical Scientist Training Program, University of Michigan Ann Arbor, MI 48109; 4 Cellular and Molecular Biology Program, University of Michigan Ann Arbor, MI 48109; 5 Howard Hughes Medical Institute, University of Michigan Ann Arbor, MI 48109.

## Abstract

Tissues are maintained in a homeostatic state by balancing the constant loss of old cells with the continued production of new cells. Tissue homeostasis can shift between high and low turnover states to cope with environmental changes such as nutrient availability. Recently, we discovered that the elimination of transit-amplifying cells plays a critical role in maintaining the stem cell population during protein starvation in the *Drosophila* testis. Here, we identify *spict*, a gene expressed specifically in differentiating cyst cells, as a regulator of spermatogonial death. Spict is upregulated in cyst cells that phagocytose dying spermatogonia. We propose that phagocytosis and subsequent clearance of dead spermatogonia, which is partly promoted by Spict, contribute to stem cell maintenance during prolonged protein starvation.

Tissue homeostasis can shift between high and low turnover states depending on environmental variables. For example, tissues can decrease turnover and/or scale down their overall size when the nutrients are limited^1^,^2^,^3^. Stem cells are widely considered the master regulators of tissue homeostasis, and how stem cells respond to changes in their external environment has been heavily studied^4^,^5^,^6^. In many tissues, however, the majority of cell proliferation occurs in transit-amplifying cells, which are stem cell progeny that divide mitotically prior to terminal differentiation to lessen the proliferative burden on stem cells^7^,^8^,^9^,^10^,^11^. Despite this, the response of transit-amplifying cells during shifting tissue homeostasis is poorly explored.

The *Drosophila* testis is an excellent model system to study the behavior of stem cells and transit-amplifying cells owing to the well-defined anatomy of the tissue and the ample genetic tools available for manipulating gene function in a cell type-specific manner. At the apical tip of the *Drosophila* testis resides two stem cell populations: germline stem cells (GSCs) and somatic cyst stem cells (CySCs). They are anchored to hub cells that organize the stem cell niche for both stem cell populations (Fig. 1A)^12^,^13^. In addition, CySCs encapsulate GSCs, and together with the hub cells, function as a part of the GSC niche by contributing to the critical signaling environment^14^,^15^. Upon stem cell division, GSCs produce gonialblasts (GBs), whereas CySCs produce cyst cells (CCs). GBs undergo four rounds of transit-amplifying divisions as spermatogonia (SGs). As cytokinesis of these divisions is incomplete, these transit-amplifying divisions yield a cluster of 16 interconnected spermatogonia (SGs), which then undergo meiotic divisions and spermiogenesis. Connectivity of SGs (2-cell, 4-cell, 8-cell, 16-cell SGs) serves as a reliable marker for their differentiation stage (Fig. 1A). Throughout this process, a pair of CCs envelop the SGs and help regulate their differentiation. CCs are critical for the survival and differentiation of SGs beyond the 2-cell SG stage (Fig. 1A)^16^.

Recently, we reported that SG death dramatically increases in response to protein starvation^17^. The GSC population, however, is relatively well maintained even during a prolonged period of protein starvation. After an initial drop in GSC number from ~8/testis to ~6/testis after 3–6 days of starvation^2^, the remaining ~6 GSCs can be stably maintained for additional ~20 days while continuing to divide at an unchanged rate compared to fed conditions^17^. This argues that transit-amplifying cells, but not stem cells, may be a major point of regulation in response to changes in nutrient conditions. We have shown that starvation-induced SG death is triggered by apoptosis of CCs^17^. When CC death is blocked by inhibiting apoptosis, starvation-induced SG death was also blocked. Concomitantly, testes failed to maintain their GSC population, leading to collapsed tissue homeostasis and compromised ability to recover upon reintroduction of nutrients^17^. These results led us to speculate that SG death upon protein starvation serves as a mechanism to protect GSCs in two ways. First, SG death would reduce the need for nutrients, thereby indirectly saving nutrients for GSCs. Second, nutrients from dead SGs may be recycled to feed GSCs. However, the underlying mechanisms to recycle nutrients from dead SG to support GSC survival and proliferation remain elusive.

Here, we report our characterization of *spichthyn (spict)*, a gene that is expressed in differentiating CCs. We find that Spict protein is specifically upregulated in CCs near dying SGs. We show that CCs phagocytose dead SGs, and that Spict is associated with lysosomes during phagocytosis of SGs, suggesting that *spict* might be involved in the process of SG phagocytosis or in the clearance of dead SGs. Finally, *spict* mutants fail to maintain the GSC population during protein starvation. Taken together, we propose that SG death is facilitated by *spict* and plays an important role in protecting the GSC population during protein starvation, possibly via recycling of nutrients from dead SGs.

## Results

### *spict* is expressed in differentiating cyst cells

**Figure 1:**
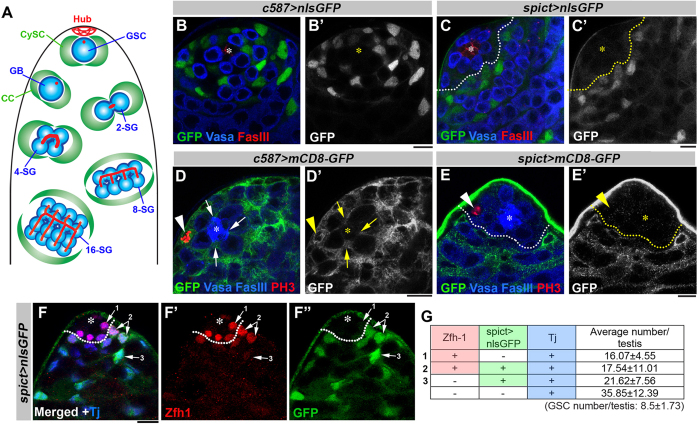
s*pict* is expressed in differentiating cyst cells. (**A**) Diagram of early spermatogenesis at the apical tip of the *Drosophila* testis. Germline stem cells (GSCs), gonialblast (GB), 2,4,8,16-cell spermatogonia (SGs), cyst stem cells (CySCs), cyst cells (CCs). GSCs and CySCs are attached to the stem cell niche component hub cells. CySCs encapsulate GSCs. GSCs produce GBs by asymmetric division. GBs are encapsulated by CCs, which promote differentiation of germ cells as SGs. (**B**,**C**) Expression of UAS-nlsGFP under the control of the *c587-gal4* driver (**B**) or the *spict-gal4* driver (**C**). nlsGFP illuminates the nuclei of gal4-expressing cells. Asterisk indicates the hub; a dotted line indicates the boundary of *spict* expression. Bar: 5 μm. (**D**,**E**) Expression of UAS-mCD8-GFP under the control of the *c587-gal4* driver (**D**) or the *spict-gal4* driver (**E**). mCD8-GFP outlines the cell surfaces of gal4-expressing cells. Processes of cyst cells are outlined by expression of membrane-bound UAS-mCD8-GFP with the pan-cyst cell driver *c587-gal4* (**D**) or *spict-gal4* (**E**). Mitotic cells are labeled with PH3 (arrowhead). CySC processes that touch the hub are indicated by arrows (**D**,**D’**). (**F**) Apical tip of a testis showing nlsGFP expression under control of the *spict-gal4* driver and co-stained with Zfh-1 (red) and Tj (blue). Tj is a marker for early CCs^35^. (**G**) Quantification of somatic cells based on the expression of Zfh-1, Tj and *spict* > *nls-GFP*. The data are shown as mean ± s.d. N = 13 testes were scored.

In a small-scale screen to identify genes expressed in the *Drosophila* testis, we identified a *gal4* enhancer trap of *spichthyin (spict*), which was originally identified as the *Drosophila* homolog of the human *NIPA1* and *ichthyin* genes^18^. When the expression pattern of *spict* was visualized by expressing *UAS-nlsGFP* (nuclear localization signal-containing GFP) with the *spict-gal4* driver, we found that GFP was specifically observed in the nuclei of differentiating CCs. Notably, nlsGFP was absent from the nuclei of somatic cells in close contact with hub cells, which most likely represent CySCs. In contrast, the well-established CC driver *c587-gal4*^19^ drove the expression of nlsGFP in all early somatic cells at the apical tip including the CySCs (Fig. 1B,C).

The lack of nlsGFP expression in CCs near the hub indicates that *spict-gal4* expression might be excluded from CySCs. To test this idea, we examined the relationship of *spict-*expressing cells with two characteristics of CySCs: 1) attachment to hub cells and 2) ability to undergo mitosis. To examine the attachment to hub cells, UAS-mCD8-GFP (a plasma membrane marker) was expressed using either *c587-gal4* or *spict-gal4*. Consistent with *c587-gal4* being expressed in all early CCs including CySCs, we observed mCD8-GFP-labeled cell processes attached to hub cells (Fig. 1D)^12^,^20^, and 100% of testes contained multiple mCD8-GFP-positive processes attached to hub cells (N = 19). In contrast, when the expression of UAS-mCD8-GFP was driven by *spict-gal4,* mCD8-GFP-positive processes were rarely associated with the hub (only <5% of testes contained hub-touching processes, N = 87). These results demonstrate that most *spict*-expressing cells do not contact the hub, suggesting that *spict-gal4*-expressing cells are not CySCs (Fig. 1E).

Next, we examined whether *spict-gal4*-expressing cells can undergo mitosis. In the *Drosophila* testis, CySCs are the only somatic cell population that undergoes mitosis^20^, and all other somatic cells are post-mitotic. To examine whether *spict-gal4*-expressing cells undergo mitoses, we labeled mitotic cells with anti-phosphorylated histone H3 (PH3) antibody. When PH3 staining was combined with *c587-gal4* > *mCD8-GFP*, 100% of mitotic somatic cells were mCD8-GFP-positive (N = 19), consistent with the notion that *c587-gal4* is expressed in CySCs. In contrast, when *spict-gal4* > *mCD8-GFP* was combined with PH3 staining, only 2.5% of all PH3-positive cells were also positive for mCD8-GFP (N = 119), supporting the idea that *spict-gal4*-expressing cells are rarely dividing. Taken together, these results strongly argue that *spict-gal4* is excluded from CySCs, and that *spict* expression marks differentiating CCs.

### *spict* expression can be used to better identify the CySC population in combination with Zfh-1

The best marker for labeling CySCs identified to date is Zfh-1^15^. Zfh-1 is a transcriptional repressor, whose function is critical for the maintenance of CySC identity. However, Zfh-1 not only marks CySCs but also their immediate daughters that have been displaced away from the hub and have initiated differentiation as CCs. Accordingly, the number of Zfh-1-positive cells is higher than the expected number of CySCs^12^,^15^. We reasoned that *spict-gal4* could be used to negatively mark CySCs and to better identify the CySC population when combined with Zfh-1. Indeed, we found that the Zfh-1-positive population can be subdivided into *spict-*negative and *spict*-positive populations (Fig. 1F). We scored the number of ‘Zfh-1-positive, *spict-*negative’ and ‘Zfh-1-positive, *spict*-positive’ somatic cells. We observed that there were 33.6 ± 10.3 Zfh-1-positive cells in the wild type flies used in this study (Fig. 1G). Among the Zfh-1-positive cells, 16.1 ± 4.6 were *spict-*negative, whereas 17.5 ± 11.0 were *spict*-positive (N = 13 testes). Given that essentially all CySC characteristics (attachment to the hub and the ability to divide) are confined within ‘Zfh-1-positive, *spict*-negative’ cells, we conclude that this population (~16 cells/testis) represents the population with the highest CySC concentration identified to date.

### *spict*-expressing CCs may become CySCs

It is well established that GSCs divide asymmetrically by orienting their mitotic spindles perpendicular to hub cells and that this spindle orientation is prepared by stereotypical centrosome positioning throughout the cell cycle^21^. In contrast, CySCs do not have consistent centrosome positioning in interphase. They enter mitosis with randomly oriented spindles, but reposition their spindles during anaphase such that one daughter of the CySC division remains attached to the hub, whereas the other daughter is displaced away from the hub and initiates differentiation^20^. Based on these observations, we proposed that CySC divisions are also stereotypically asymmetric. However, a recent lineage tracing study found that CySCs likely undergo stochastic self-renewal and differentiation and suggested that there is no stereotypical asymmetric stem cell division in the CySC population^22^.

**Figure 2:**
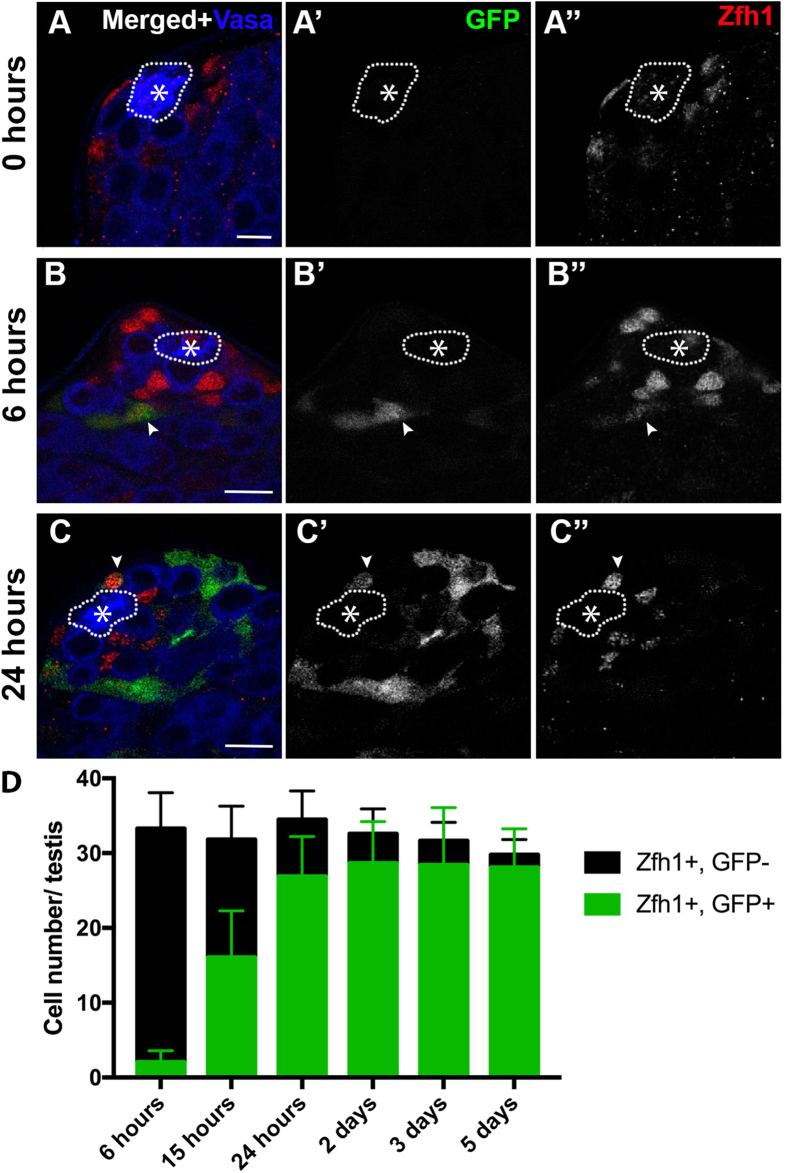
Lineage tracing of *spict*-expressing cells in the testis reveals the frequent conversion of *spict*-positive cells into CySCs. (**A**–**C**) *spict-gal4; UAS-FLP* flies were crossed with *Act* > *stop* > *gal4, UAS-EGFP; tubP-gal80*^*ts*^ at the permissive temperature (18 °C) until eclosion (A, 0 hours). Upon eclosion, adult flies were transferred to the non-permissive temperature (29 °C) for 6 hours (**B**), and 24 hours (**C**). Zfh-1-positive, GFP-positive CCs (arrowheads) appear after 6 hours, and Zfh-1-positive, GFP-positive CySCs appear after 24 hours (**C**). Bar: 10 μm. The hub is indicated by an asterisk and outlined by a dotted line. (**D**) Quantification of Zfh-1-positive cells based on GFP expression. The data are shown as mean ± s.d. N > 15 testes were scored for each time point.

We reasoned that *spict-gal4*′s ability to specifically mark differentiating CCs might help to reconcile these differences. We used *spict-gal4* to drive the expression of FLP recombinase to permanently label CCs that are *spict*-positive at any point (*spict-gal4, UAS-FLP, act* > *stop* > *gal4, UAS-GFP, tubP-gal80*^*ts*^) and followed their fates. The flies were raised at 18 °C to repress *spict-gal4* expression until adulthood, and the young adult flies of this genotype were shifted to 29 °C after eclosion to allow the lineage tracing of *spict*-positive cells. Before temperature shift, there was no *gal4* activity, thus the entire testis was GFP-negative (Fig. 2A). 6 hours after temperature shift, GFP-positive, Zfh-1-positive cells were apparent (Fig. 2B). At this point, none of GFP-positive, Zfh-1-positive cells were observed adjacent to the hub cells, consistent with the above results that *spict*-positive cells are not CySCs. By 24 hours after temperature shift, the number of GFP-positive Zfh-1-positive cells had dramatically increased, and they began to appear adjacent to the hub (Fig. 2C,D). Eventually, almost all Zfh-1-positive cells became GFP-positive (Fig. 2D). This suggests that *spict*-positive CCs frequently revert back to a *spict*-negative, CySC state. Although we cannot completely exclude the possibility that *spict-gal4* is weakly expressed in CySCs (at a undetectable level), resulting in lineage-marking of CySCs, the fact that the initial lineage-marked cells always appear as CCs indicates that most of lineage-marking events occur in CCs, rather than CySCs. The frequent reversion of *spict*-positive CCs to *spict*-negative CySCs is consistent with the neutral competition model proposed by Amoyel *et al*.^22^. If CySCs divide symmetrically without ever expressing *spict*, our *spict-gal4*-mediated lineage tracing would not have resulted in an increase of GFP-positive CySCs. Combined with our previous report that CySCs consistently divide asymmetrically with regard to the attachment to the hub cells (i.e. 96% of CySC division yielded one daughter cell attaching to the hub, the other detaching from the hub)^20^, we propose that CySCs divide asymmetrically with respect to *spict* expression (and attachment to the hub cells), generating a *spict*-negative CySC and a *spict*-positive differentiating CC, and that *spict*-expressing CCs frequently dedifferentiate to reacquire CySC identity. As a result of frequent dedifferentiation events, the CySC population follows a neutral competition model as demonstrated by Amoyel *et al*.^22^. In the face of frequent dedifferentiation, which agrees well with the study by Amoyel *et al*., it is unclear why CySCs nevertheless undergo asymmetric divisions with respect to hub cell attachment and *spict* expression.

### *spict* does not regulate BMP signaling in the *Drosophila* testis

It was shown that *spict* facilitates BMP receptor endocytosis to negatively regulate BMP activity in the *Drosophila* neuromuscular junction^18^. Spict bears homology to mammalian NIPA1, 2 and Ichthyin proteins, which are predicted to encode transmembrane proteins^23^,^24^,^25^. NIPA1 was also shown to be required for downregulating BMP signaling in the cultured neuron^26^,^27^. Therefore, we sought to test whether *spict* may be required for BMP signaling in the *Drosophila* testis.

Upon BMP ligand-receptor binding, Mad (mother against Dpp) is phosphorylated (pMad) and translocates to the nucleus for downstream transcriptional regulation^28^,^29^,^30^. By using pMad as a readout of BMP activity, we investigated whether a *spict* mutant may have altered BMP signaling activity in the testis. In wild type testes, it is well known that GSCs are positive for pMad, as BMP signaling functions in GSC self-renewal^14^,^31^,^32^. In addition, it has been observed that differentiating CCs far from the hub also show pMad signal, which was shown to regulate spermatocyte differentiation^33^,^34^. We observed no detectable difference in pMad levels between control and *spict* mutant testes, either in the GSC population or in the late CC population (Supplementary Fig. S1). These results suggest that *spict* might not play a role in regulating BMP activity during spermatogenesis.

### *spict* is required for SG death and maintenance of the GSC pool upon starvation

**Figure 3:**
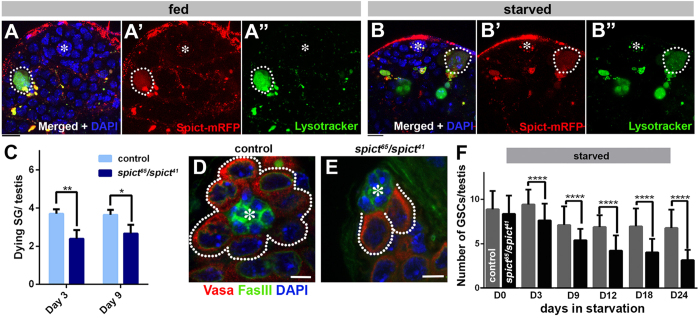
s*pict* is required for SG death and GSC maintenance during protein starvation. (**A**,**B**) Localization of Spict-mRFP (*spict-gal4* > *UAS-spict-mRFP*) in an adult testis under fed (**A**) and protein-starved conditions (**B**). A dotted line encircles dying SG; Bar: 10 μm. (**C**) Quantification of SG death in control (*spict*^*41*^*/CyO*) and mutant (*spict*^*65*^*/spict*^*41*^) testes upon 3 and 9 days of protein starvation. The data are shown as mean ± s.d. N > 120 testes were scored for each data point. *P < 0.05, **P < 0.005 (Student’s t-test, two-tailed). (**D**,**E**) Examples of the apical tip after 24 days of protein starvation in control (**D**) and *spict*^*65*^*/spict*^*41*^mutant (**E**) testes. GSCs are indicated by dotted lines. The hub is indicated by asterisks. Bar: 5 μm. (**F**) GSC number in control (gray) and *spict*^*65*^*/spict*^*41*^mutant (black bar) testes during protein starvation. The data are shown as mean ± s.d. N = 15 testes were scored for each data point. (****P < 0.00005, Student’s t-test, two-tailed).

In order to explore the function of *spict* in *Drosophila* spermatogenesis, we examined Spict protein localization using *UAS-spict-mRFP* expressed under the control of *spict-gal4* (*spict-gal4* > *spict-mRFP. UAS-spict-mRFP* was shown to complement *spict* mutant phenotypes)^18^. We noticed that Spict-mRFP extensively overlapped with Lysotracker staining (Fig. 3A,B, see below for detailed Spict localization), which we previously showed to mark dying SGs^17^. These observations prompted us to ask whether *spict* plays a role in SG death.

In our previous work, we showed that SG death is significantly upregulated upon protein starvation^17^. SG death is triggered by the apoptosis of an encapsulating CC, which in turn initiates SG death, likely due to the CCs’ essential role in SG survival^16^. We further showed that inhibition of SG death by blocking CC apoptosis led to a continuous decline in GSC number and an eventual collapse in tissue homeostasis under protein starvation conditions, suggesting that eliminating SGs plays a critical role in maintaining the GSC population during starvation^17^. Spict-mRFP was observed in dying SGs irrespective of nutrient conditions (Fig. 3A,B). We found that SG death was significantly decreased in *spict* mutants (*spict*^*65*^*/spict*^*41*^) or upon RNAi-mediated knockdown of *spict* in CC lineage using CC-specific driver *tj-gal4* (*tj-gal4* > *spict*^*RNAi*^)^35^ compared to control (Fig. 3C, Supplementary Fig. S2). Although we cannot exclude the possibility that *spict* may also play a role in germ cells, CC-specific expression pattern of *spict* (Fig. 1) and the fact that both *spict* loss-of-function mutant as well as CC-specific knockdown of *spict* (*tj-gal4* > *spict*^*RNAi*^) result in a similar phenotype (Fig. 3C, Supplementary Fig. S2) argue that *spict* functions mainly in CCs. Consistent with our previous study, which showed that SG death is required for maintaining the GSC pool^17^, *spict* mutants failed to maintain GSC number during prolonged protein starvation (Fig. 3D–F). *spict* mutants maintained similar numbers of GSCs compared to control under fed conditions (data not shown), suggesting that *spict*’s requirement is more profound during protein starvation.

Taken together, these results suggest that *spict* is required to promote starvation-induced CC/SG death, and consequently for the maintenance of the GSC pool in response to protein starvation.

### Spict protein is expressed in CCs associated with dying SGs

**Figure 4:**
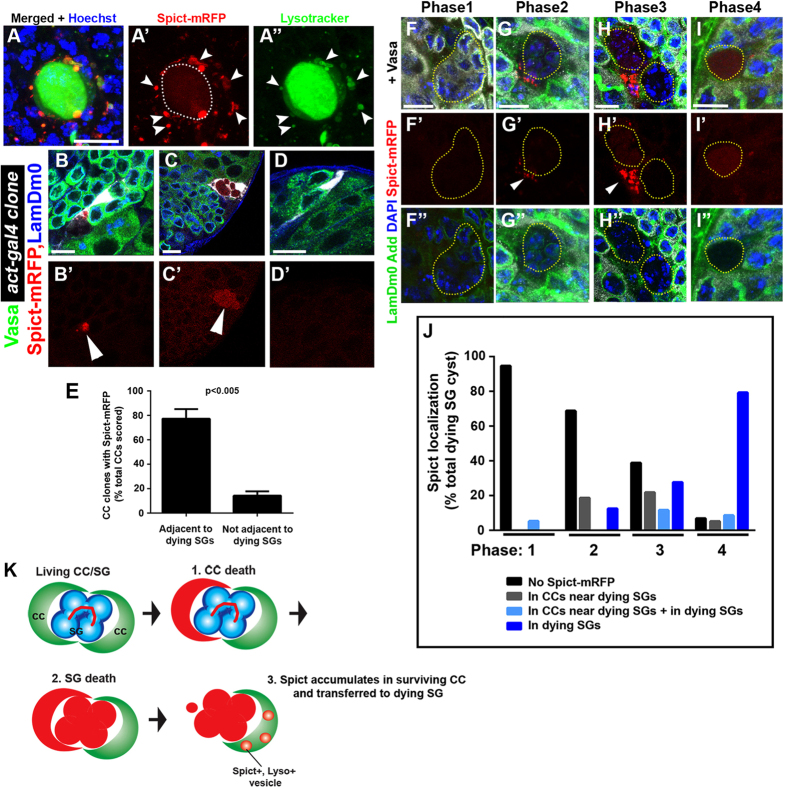
Spict localizes to CCs associated with dying SG and transferred to dying SGs. (**A**) An example of dying SGs positive for Lysotracker (green) and Spict-mRFP (red) in a *spict-gal4* > *UAS-spict-mRFP* testis. Bar: 10 μm. (**B**–**D**) Examples of CC clones that express Spict-mRFP in the presence (**G**,**H**) or absence (**I**) of neighboring dying SGs. Even though all CC clones (*act-gal4*-positive, white) activate *UAS-spict-mRFP* expression, Spict-mRFP protein was visible only when the clones were adjacent to dying SGs. Arrowheads indicate dying SGs (**G’,H’**). Bar: 10 μm. (**E**) Quantification of the percentage of Spict-mRFP-positive CC clones in the presence or absence of neighboring dying SGs. The data are shown as mean ± s.d. N > 60 clones were scored. p-value (Student’s t-test, two tailed) is provided. (**F**–**I**) Representative images of Spict-mRFP localization during the course of SG death. Phase 1(**B**), phase 2 (**C**), phase 3 (**D**) and Phase 4 (**E**). Yellow dotted lines encircle the dying SGs. Arrowheads indicate CCs with upregulated Spict-mRFP near the dying SGs (**C’,D’**). Bar: 10 μm. (**J**) Distribution of Spict-mRFP localization during the course of SG death. (**K**) Model of the SG death process: the living SGs are encapsulated by a pair of CCs. The death of one CC (1) triggers SG death (2) and the Spict protein accumulates in the surviving CC and is transferred to the dying SGs (3).

To gain further insights into the role of *spict* in SG death, we expanded our analysis of Spict localization. Spict protein was highly upregulated in CCs near dying SGs and appeared punctate in those cells (Fig. 4A). Spict-mRFP accumulation near dying SGs is likely due to increased mRNA translation or protein accumulation as the *spict-gal4* reporter (*spict-gal4* > *UAS-GFP*) (Fig. 1) shows ubiquitous expression of GFP in early CCs (excluding CySCs). In support of this idea, when a CC clone expressing *UAS-spict-mRFP* was generated using a constitutive *actin* promoter (*hs-Flp, Act* > *stop* > *gal4, UAS-GFP, UAS-spict-mRFP*), Spict-mRFP was observed specifically when the CC clone was adjacent to dying SGs (Fig. 4B–D): Accumulation of Spict-mRFP was observed in 77% of the CC clones that are associated with dying SGs, whereas Spict-mRFP accumulation was observed only in 14% of the CC clones that are not adjacent to dying SGs (Fig. 4E). Because Spict-mRFP does not accumulate unless associated with the dying SGs, it is unlikely that the use of spict-gal4 causes dominant, over-expression phenotype. Consistently, we did not observe any detectable changes in the GSC number or the frequency of SG death in Spict-mRFP-expressing testis (*spict-gal4* > *spict-mRFP*).

In addition to accumulation in CCs near the dying SGs, Spict-mRFP was observed in the dying SGs themselves (Fig. 4A). Since *spict-gal4*, which is expressed in CCs but not in germ cells (Fig. 1), was used to drive *UAS-spict-mRFP* expression, Spict-mRFP might be transferred from the CC to the dying SG. A similar result was obtained using a well-established CC driver, *tj-gal4* (Supplementary Fig. S3), suggesting that the Spict-mRFP observed in dying SGs is not due to upregulation of *spict* transcription in dying SGs.

To better understand Spict-mRFP’s localization during the progression of SG death, we first conducted a detailed characterization of the SG death process by combining various markers (Vasa, LaminDm0, DAPI and Lysotracker, Supplementary Fig. S4), extending our previous characterization^17^. Based on these markers, we now divided SG death into four phases. During phase 1, as SGs initiate cell death, the level of Vasa (a germ cell marker) decreases compared to neighboring SGs that are not dying, coinciding with the appearance of Lysotracker in the dying SG at a very low level. Lysotracker intensity increases significantly during phase 2, indicating acidification of the germ cell cyst and thus progression of the cell death process. This phase is also characterized by the complete disappearance of Vasa staining, whereas nuclear envelope marker LaminDm0 and DAPI staining remains distinct at this phase, suggesting that nuclear compartment is still relatively intact. This is followed by phase 3, when LaminDm0 staining disappears, while DAPI staining remains, indicating that digestion of nuclear compartment is progressing. Finally during phase 4, all Vasa, LaminDm0 and DAPI staining disappears, and the remnants of dead SGs are visible only with Lysotracker, which sometimes becomes weaker than phases 2 and 3. The temporal order of SG death was confirmed by inducing synchronized SG death through expression of the proapoptotic gene *grim* in CCs, as described previously^16^. Indeed, after induction of *grim* expression, phases 1 through 4 appeared in the expected order, suggesting that phase 1–4 characterization is accurate. (Supplementary Fig. S4C).

We adapted these criteria to further characterize Spict localization in detail during SG death (Fig. 4F–J). Because the patterns of Vasa, LaminDm0 and DAPI during SG death were fairly consistent, cell death phases can be determined even without the use of Lysotracker. Thus, we combined Spict-mRFP with Vasa, LaminDm0, and DAPI to follow the localization of Spict-mRFP during SG death. We found that dying SGs are not yet associated with Spict-mRFP during phase 1 (Fig. 4F). During phase 2, Spict-mRFP starts to accumulate near dying SGs (Fig. 4G), followed by phase 3, when Spict-mRFP is highly upregulated in the CCs near dying SGs and is also observed in dying SGs (Fig. 4H). During phase 4, Spict-mRFP in the CC is mostly gone and is mainly observed in dying SGs (Fig. 4I).

The above results suggest that Spict-mRFP protein accumulates in CCs and dying SGs after the initiation of SG death, i.e. in phases 2 through 4. This raised the question regarding in which CCs Spict-mRFP might be upregulated. Our previous study showed that SG death is triggered by the apoptosis of CCs^17^, indicating that CC apoptosis must occur before/during phase 1. If this is the case, Spict-mRFP is not expressed in the CCs that apoptose to trigger SG death. We speculate that only one of two CCs that encapsulate the SG cyst dies to trigger SG death, and the other CC might survive and upregulate Spict protein. Alternatively, it is possible that other CCs, such as those encapsulating other (alive) SGs, might upregulate Spict protein. However, we do not believe the latter possibility likely, as we have never observed CC clones that encapsulate multiple SG clusters, including living SGs and dying SGs, in our various experiments in the course of this study. Irrespective of its origin, dying SGs are clearly encapsulated by CCs with high Spict expression (Fig. 4B–E). Taken together, we propose that the death of SGs is triggered by apoptosis of encapsulating CC, and that Spict is subsequently upregulated in the neighboring, surviving CC (Fig. 4K).

### Spict localizes to the late endosome/phagosome during engulfment of dying SGs by CCs

**Figure 5:**
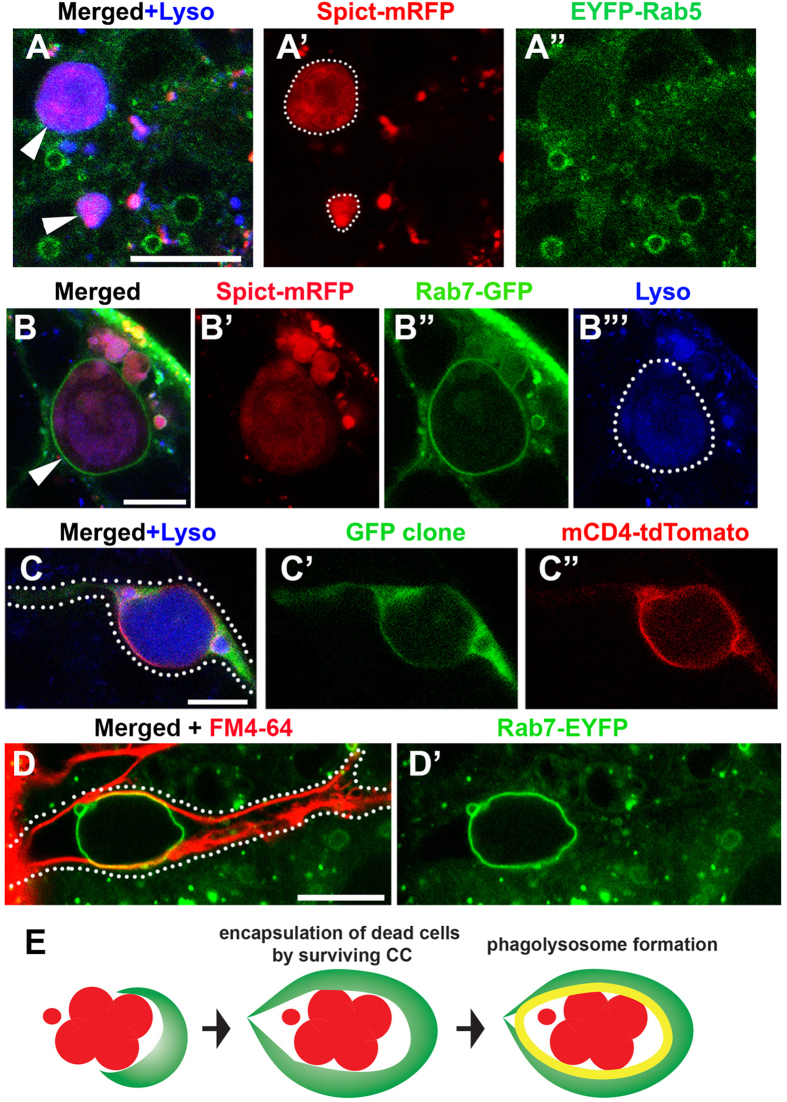
Spict localizes to Rab7-positive phagosomes that encapsulate dying SGs. (**A**) Spict-mRFP expressed in CCs (*spict-gal4* > *UAS-spict-mRFP*) does not colocalize with the early endosome marker Rab5. Dying SGs are indicated by arrowheads. Bar: 10 μm. (**B**) The late endosome marker Rab7 colocalizes with Spict-mRFP and forms a large vesicle encapsulating dying SGs (arrowhead). Dying SG is encircled by dotted line in B”’. Bar: 5 μm. (**C**) An example of a single CC clone expressing GFP and mCD4-tdTomato (*hs-FLP, act* > *stop* > *gal4, UAS-GFP, UAS-mCD4-tdTomato*) encapsulating dying SGs entirely. A single CC clone is indicated by dotted line. Bar: 10 μm. (**D**) Rab7-EYFP testis stained for the membrane dye FM4-64 to label the CC plasma membrane, demonstrating that the Rab7-positive vesicle is entirely encapsulated within a single CC. CC boundary is indicated by dotted line. Bar: 10 μm. (**E**) Model of SG phagocytosis by the surviving CC.

Previously, it was shown that Spict colocalizes with Rab5-positive early endosomes^18^. Likewise, it was also shown that a mammalian homologue of Spict, NIPA1, colocalizes with various endosomal compartments^36^. Their localization to endosomal compartments was linked to their role in the regulation of BMP receptors. Although our results indicate that *spict* is likely not regulating BMP signaling in the *Drosophila* testis (Supplementary Fig. S1), colocalization of Spict-mRFP with Lysotracker (Figs 3 and 4) indicates that Spict is involved in the endocytic pathway. Thus, we examined the potential colocalization of Spict-mRFP with various endosomal compartments by expressing Spict-mRFP together with EYFP-tagged endogenous Rab small GTPases^37^, or UAS-GFP tagged Rab GTPases^38^,^39^ using *spict-gal4*. We did not observe obvious colocalization of Spict-mRFP with early endosomal markers, such as Rab5, or with recycling endosome markers, such as Rab4, Rab11 and Rab35 in the testis (Fig. 5A and Supplementary Fig. S5)^40^. However, we found that Spict often colocalizes with Rab7, a marker for the late endosome (Fig. 5B, 68% of dying SG cyst was encapsulated by Rab7-positive vesicle, N = 183 dying SG cysts in 51 testes scored). Given that Spict also colocalizes with Lysosomes, it appears that Spict is associated with the late endosome/lysosome compartment in the *Drosophila* testis.

While studying the colocalization, we noticed that Spict-positive dying SGs were frequently contained within a large vesicle positive for Rab7-EYFP (Fig. 5B). Such Rab7-positive ‘vesicles’ could reach almost ~20 μm in diameter, which is the size of the entire cyst of dying SGs containing up to 16 cells. Since Rab7 is known to be required for phagosome maturation^41^,^42^, we suspected that the entire dying SG might be phagocytosed by the neighboring cells (i.e. CCs). Indeed, when a single CC clone expressing GFP and a membrane marker (mCD4-tdTomato) (*hs-FLP; act* > *stop* > *gal4, UAS-mCD4-tdTomato*) was induced, the plasma membrane of the clone marked by mCD4-tdTomato continuously wrapped around the Lysotracker-positive dying SGs (Fig. 5C). This CC is most likely the surviving CC that does not undergo apoptosis to initiate SG death. Moreover, staining with another membrane dye, FM4–64, corroborated that the Rab7-EYFP-positive vesicle was contained within a single CC (Fig. 5D). Taken together, these data suggest that dying SGs are encapsulated by a single CC, likely via phagocytosis (Fig. 5E).

### *spict* is required for normal progression of SG death

**Figure 6:**
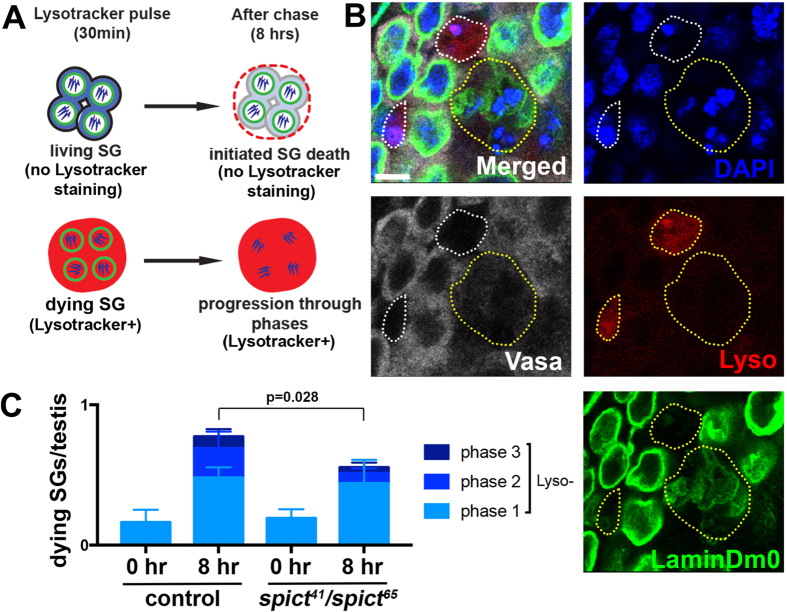
s*pict* regulates the progression of SG death. (**A**) Schematic of the Lysotracker pulse-chase experiment. The dissected testes were stained with Lysotracker for 30 minutes and cultured in lysotracker-free media for 8 hours. SGs that initiated death during the 8 hour culture period are Lysotracker-negative, whereas SGs that were already dying at the beginning of culture are Lysotracker-positive. Dying SGs were identified by DAPI, Vasa and LaminDm0 staining. (**B**) Example of SGs that initiated the death process during the chase period (Lysotracker-negative, yellow dotted line) and those that were already dying during pulse period (Lysotracker-positive, white dotted lines). Bar: 5 μm. (**C**) Quantification of cell death phase for SGs that committed to death during the chase period (lysotracker-negative). The data are shown as mean ± s.d. N > 90 testes were scored for each genotype. p-value (Student’s t-test, two tailed) is provided.

Based on our observation that Spict is highly upregulated in the CCs that phagocytose dying SGs, we speculated that *spict* might be required for processing/clearing the dead SGs. However, we did not see obvious differences between control and *spict* mutants in the degree of acidification of the dying SG (not shown). Moreover, although overall SG death was decreased in *spict* mutant as described above (Fig. 3C), once SGs initiate death process, the frequency of dying SGs being encapsulated by Rab7-positive phagosome or not was not different between control and *spict* mutant: 68% of dying SG cysts was encapsulated by Rab7-positive vesicle (N = 183 dying SG cysts) in control, 84% of dying SG cysts was encapsulated by Rab7-positive vesicle (N = 204 dying SG cysts from 66 testes scored) in *spict*^*65*^*/spict*^*41*^ mutant. The SG cell death process from initiation to completion appears to take a long time (>24 hours, based on indirect inference from our observations), making it difficult to assess the cell death process via live observation. Therefore, to study the process of SG death progression, we developed an *ex vivo* testis culture system: first, dissected testes were stained with Lysotracker for 30 minutes to label SGs that were already dying. Testes were then transferred to Lysotracker-free medium and cultured for 8 hours (Fig. 6A). At the end of the culture period, testes were fixed and stained for Vasa, LamDm0, and DAPI to identify dying SGs as described above (Supplementary Fig. S4). With this method, SGs that were already dying before the culture period can be identified as Lysotracker-positive dying SGs, whereas SGs that initiated the cell death process during the culture period can be identified as Lysotracker-negative dying SGs (Fig. 6B). We restricted our analysis to the Lysotracker-negative dying SGs, because these allowed us to precisely follow how SGs initiated the death process and progressed through the cell death stages during the 8-hour chase period.

Using this approach, we compared control and *spict* mutant testes to see whether *spict* mutants might show any defects in the process of SG death progression. After the 8-hour chase period, control testes had 0.77 ± 0.15 Lysotracker-negative dying SGs/testis (N = 119), whereas *spict* mutant testes had 0.55 ± 0.16 dying SGs/testis (N = 123 testes, p = 0.028). This result suggests that *spict* mutants are defective in initiating SG death, which is consistent with the results described in Fig. 3. In addition, 38% of dying SGs in control testes were observed to be in phase 2 and 3, indicating that some dying SGs in control testes progressed through the SG death phases after initiation during the 8-hour culture period (Fig. 6C). In contrast, in *spict* mutant testes, only 21% of Lysotracker- negative dying SGs progressed beyond phase 1 (p = 0.032). These results indicate that *spict* mutants are also defective in progressing through the SG death phases after the initiation of death (Fig. 6C). This further indicates that it takes longer for SGs to complete the cell death process in *spict* mutants. If it takes longer for each dying SG to complete the death process in *spict* mutants, the frequency of SG death in *spict* mutants is likely an overestimate, when estimated based on the number of dying SGs in a fixed sample (Fig. 3C). Thus, the decrease in SG death in *spict* mutants described above is likely more profound than is shown in Fig. 3C. In summary, we conclude that *spict* is required for initiation and progression of SG death in the *Drosophila* testis.

## Discussion

In this study, we identified *spict* as a gene that is specifically expressed in the differentiating cyst cells (CCs) of the *Drosophila* testis and that it can serve as a novel marker to better identify cyst stem cells (CySCs). We showed that Spict protein is specifically stabilized in the CCs that envelope dying SGs, and that *spict* is required for the normal progression of SG death. Our study indicates that CC-mediated SG death plays a critical role in allowing the tissue to cope with protein starvation in order to maintain the germline stem cell (GSC) pool.

Our data show that dying SGs are encapsulated entirely by Rab7-positive vesicles, and these vesicles are contained within a single CC. Thus, we propose that dying SGs are phagocytosed by neighboring CCs. Our earlier study showed that the apoptosis of a CC is required to trigger SG death^17^. Now, our study suggests that the remaining CC encapsulates the dying SGs, further elucidating how SG death occurs in the *Drosophila* testis. We propose the following scenario of CC-mediated SG death: first, one of the two CCs that encapsulate the SGs undergoes apoptosis in response to certain stimuli, such as protein starvation. Death of one CC breaks the ‘blood-testis-barrier’ generated by the pair of CCs^43^ and leads to SG death^16^. The remaining CC (‘surviving CC’), or possibly another neighboring CC, now engulfs the dying CC and the SGs to clear the dead cells via phagocytosis. Spict protein was specifically upregulated in surviving CCs and apparently transferred to dying SGs. Considering that the progression of SG death is slower in *spict* mutants compared to the control, we speculate that *spict* may be required for the progression of phagocytosis, the subsequent digestion of engulfed dead cells, and/or the recycling of digested SG material in the surviving CCs. However, we did not observe any differences in the frequency of large, Rab7-positive phagosome formation between control and *spict* mutant testes. In addition, we did not detect any differences in the degree of lysosome/phagosome acidification in control vs. *spict* mutant testes. Therefore, it remains unclear exactly how *spict* may promote the progression of SG death.

Programmed cell death can be induced in a cell-intrinsic (suicide) or -extrinsic (murder) manner^44^. Recently, engulfment genes were shown to be required for the developmentally programmed death of the B.alapaav cell in *C. elegans* by assisting with the cell death processes^45^. In addition, engulfment genes were also shown to promote the unequal segregation of apoptotic potential to induce NSMsc cell death in *C. elegans*^46^. Likewise, it was shown that in *Drosophila* oogenesis, the phagocytic machinery of follicle cells is required for developmentally programmed death and removal of nurse cells^47^. Starvation-induced SG death in the *Drosophila* testis described in this and our previous study^17^ holds a striking similarity with these cell death processes in that the cell death precedes via a complex interaction of multiple cell types. SG death depends on the apoptosis of a CC, and later, its removal/clearance depends on the phagocytic activity of the remaining CC. Our data suggest that *spict* is likely required for promoting the process of SG removal after phagocytoses by the remaining CC.

In summary, our study identified *spict* as a gene expressed in the differentiating CCs in the *Drosophila* testis. *spict* is required for the proper progression of SG death and for maintaining the GSC population during protein starvation. Through a detailed characterization of Spict localization, we revealed a highly regulated process of SG death that involves CC death and phagocytosis by the surviving CC, and our data suggest that *spict* may be involved in this process. We propose that carefully regulating the death of transit-amplifying cells during starvation is a critical mechanism to preserve the stem cell population and that the transit-amplifying cell population serves as a major point of regulation in shifting tissue homeostasis.

## Materials and Methods

### Fly strains and husbandry

Flies were cultured in standard Bloomington medium at 25 **°**C. For protein starvation experiments, newly eclosed adult flies were transferred within 24 hours (day 0) onto either standard food (fed) or 16% sucrose/0.7% agar (starved) at a density of 20–40 flies per vial. Flies were transferred to fresh vials every three days. The following fly stocks were used: *c587-gal4*^19^, *nos-gal4*^48^. *UAS-spict-mRFP, spict*^41^ were generous gifts of Cahir O’Kane^18^. *UASt-H1-YFP* was a kind gift of Alexei Tulin^49^, *tj-gal4* was obtained from Xin Chen^35^. *UAS-grim* was obtained from Margaret T. Fuller^50^. *UAS-mCD4-tdTomato, UAS-spict-RNAi*^*GLC01402*^*, UAS-spict-RNAi*^*HMS01647*^*, UAS-Rab4-GFP, Rab5-EYFP, Rab7-EYFP, UAS-Rab7-GFP, tub-gal80*^*ts*^ were obtained from the Bloomington Stock Center. *spict-gal4*^*NP112900*^ was obtained from the Kyoto Stock Center.

### Generation of *spict* ^*65*^ allele

Two target sequences (AACAGAGC|AAGTGAGTCATA AGG and GCAAGGGA|TGTAACTAGACC TGG) for CRISPR-mediated knockout were selected to delete the whole second exon of *spict*. These constructs were co-injected into *vas-Cas9*^*ZH-2A*^ flies. The genotype of potential mutants was determined by PCR and sequenced to confirm the deletion. *spict*^*65*^ contains the deletion with imprecise repair at the junction resulting in a short, in-frame insertion (underlined, CAGAAC-AGAAACAGA-ACA) and internal deletion of *spict*.

### Immunofluorescent staining and microscopy

Immunofluorescent staining of testes was performed as described previously^51^. Briefly, testes were dissected in PBS, transferred to 4% formaldehyde in PBS and fixed for 30–60 minutes. The testes were then washed in PBS-T (PBS containing 0.1% Triton-X) for at least 30 minutes, followed by incubation with primary antibody in 3% bovine serum albumin (BSA) in PBS-T at 4 °C overnight. Samples were washed for 60 minutes (three 20-minute washes) in PBS-T, incubated with secondary antibody in 3% BSA in PBS-T at 4 °C overnight, washed as above, and mounted in VECTASHIELD with DAPI (Vector Labs). The following primary antibodies were used: mouse anti-Adducin-like (*hu-li tai shao* – Fly Base) [1:20; Developmental Studies Hybridoma Bank (DSHB); developed by H.D. Lipshitz], rat anti-vasa (1:50; DSHB; developed by A. Spradling), rabbit anti-vasa (1:200; d-26; Santa Cruz Biotechnology), mouse anti-Fasciclin III (1:200; DSHB; developed by C. Goodman), anti-LaminDm0 (1:200; DSHB; developed by P. A. Fisher), rabbit anti-Thr3-phsophorylated Histone H3 (PH3) (1:200; Upstate, Millipore, Billerica, CA), guinea pig anti-Traffic jam (Tj) (1:400; a kind gift of Dorothea Godt) (Li *et al*., 2003), rabbit anti-Zfh-1 (1:5000; a kind gift of Ruth Lehmann), rabbit anti-pMad [1:200; Abcam anti-Smad3 (Phospho S423 + S425)]. Images were taken using Leica TCS SP5 and TCS SP8 confocal microscopes with 63× oil-immersion objectives (NA = 1.4) and processed using Adobe Photoshop software. For detection of germ cell death, testes were stained with Lysotracker in PBS (1:1000) for 30 minutes prior to formaldehyde fixation. Cell death phases were identified by using anti-Vasa, anti-LaminDm0, DAPI staining and Lysotracker.

For observation of unfixed testes, testes were dissected directly into PBS and incubated in the dark with the desired dye(s) for 5 minutes, mounted on slides with PBS and imaged within 10 minutes of dissection. The dyes used in live imaging are: Lysotracker Red DND-99 (1:1000), Lysotracker Blue DND-22 or Lysotracker Green DND-26 (1:200) (Thermo Fisher Scientific), Hoechst 33342 (1:1000), and the FM4-64FX in PBS (1:200) (Thermo Fisher Scientific).

### Lineage tracing of *spict*-expressing cells

To lineage-label cells that once expressed *spict, spict-gal4* was used to drive *UAS-FLP*, which removes the stop codon between the *actin* promoter and *gal4* (*spict-gal4, UAS-FLP, Act* > *stop* > *gal4, UAS-GFP, tubP-gal80*^*ts*^). Cells that once expressed *spict* will be labeled with GFP. *spict-gal4* activity was repressed by *tubP-gal80*^*ts*^ when flies are cultured at 18 °C, ensuring that no labeling occurred before adulthood. Newly eclosed flies were shifted to 29 °C to activate *spict-gal4* expression.

### Pulse-chase experiment to track SG death progression

Freshly eclosed control (*spict*^*65*^*/CyO* or *spict*^*41*^*/CyO*) and *spict* mutant (*spict*^*64*^*/spict*^*41*^) flies were starved for three days. Testes were dissected directly into Schneider’s *Drosophila* medium (Thermo Fisher Scientific) on a glass dissection dish and soaked in Lysotracker Red (1:1000 in Schneider’s media) in the dark for 30 minutes (tubes were rotated to ensure even staining). The tissues were then rinsed with media three times prior to the chase period to avoid carry over of extra Lysotracker. <5 testis were transferred into 20 μl-droplets of media on a 35 mm petri dish. To prevent evaporation, extra media droplets were added on the dish near the tissue-containing droplets, and the dish was sealed with the parafilm. The samples were then kept in dark at room temperature for 8 hours (chase period) prior to fixation and staining.

## Additional Information

**How to cite this article:** Chiang, A. C.-Y. *et al*. *spict*, a cyst cell-specific gene, regulates starvation-induced spermatogonial cell death in the *Drosophila* testis. *Sci. Rep.* **7**, 40245; doi: 10.1038/srep40245 (2017).

**Publisher's note:** Springer Nature remains neutral with regard to jurisdictional claims in published maps and institutional affiliations.

## Supplementary Material

Supplementary Figures
