# Supplementary material for: spict, a cyst cell-specific gene, regulates starvation-induced spermatogonial cell death in the Drosophila testis
Source: Sci Rep. 2017 Jan 10;7:40245. doi: 10.1038/srep40245 (PMC5223112; doi:10.1038/srep40245)
Supplement: Supplementary Figures [file srep40245-s1.pdf]

Supplementary information

spict , a cyst cell-specific gene, regulates starvation-induced spermatogonial cell death in the _Drosophila_ testis.

Ason C.-Y. Chiang, Heiko Yang[2, 3, 4], and Yukiko M. Yamashita[1, 2, 4, 5, *]

[1]Department of Cell and Developmental Biology, Medical School

[2] Life Sciences Institute

[3] Medical Scientist Training Program

[4] Cellular and Molecular Biology Program

[5]Howard Hughes Medical Institute,

University of Michigan Ann Arbor, MI 48109

* corresponding author: yukikomy@umich.edu

Supplementary Figures

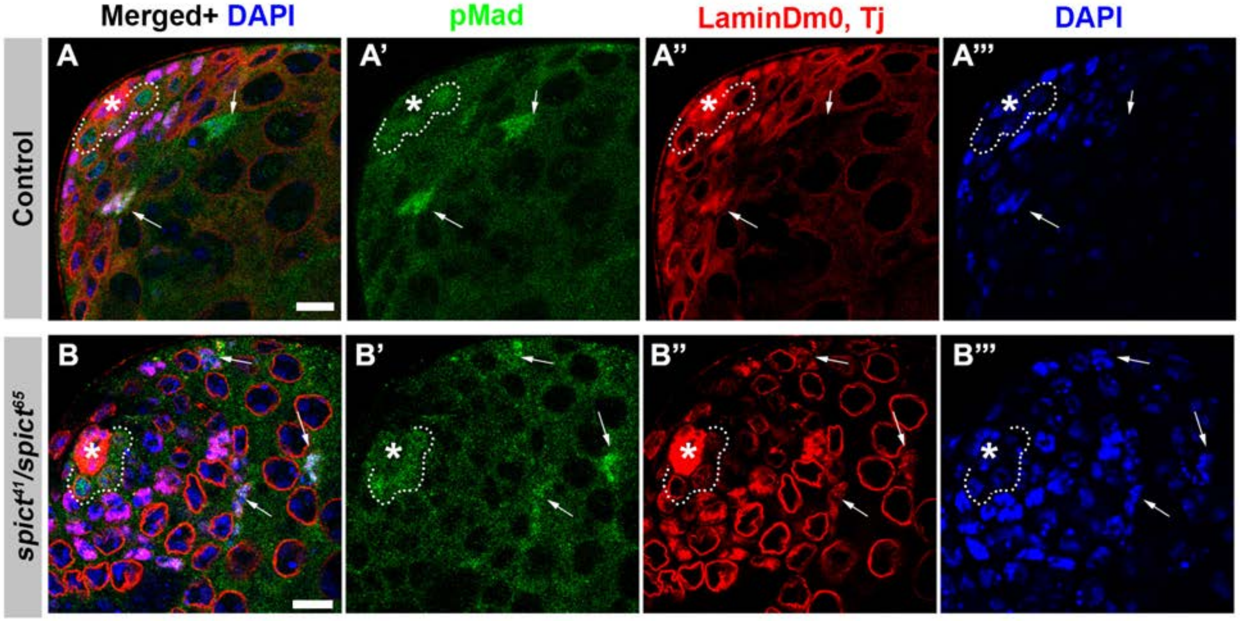

Supplementary Fig. S1. pMad level is not obviously changed in the *spict* mutant testis.

(A, B) Testes from control (*spict*^{41}/*Cyo,* A) or mutant (*spict*^{65}/ *spict*^{41}, B) testes were starved for three days upon eclosion and stained for pMad (green), LaminDm0/Tj (red) and DAPI (blue). In both genotypes, pMad was detected in the GSC nuclei (encircled by a dotted line) around the hub (asterisk) as well as late differentiating CCs (arrows) associated with spermatocytes. Bar: 10µm. N>15 testes were examined for each genotype.

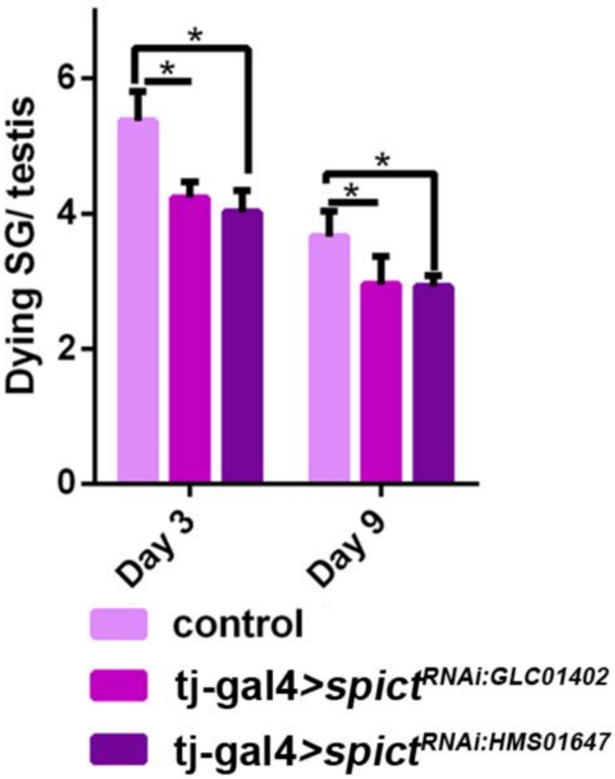

Supplementary Fig. S2. ***spict^{RNAi}*** **in CCs result in a decrease in SG death in response to protein starvation.**
Knockdown of *spict* specifically in somatic cells (*tj-gal4>UAS-spict^{RNAi}*) results in a decrease in SG death upon starvation. The data are expressed as means ± s. d. * indicates $p<0.05$ (Student's t-test, two-tailed). N>90 (triplicates of N>30) testes were scored for each data point.

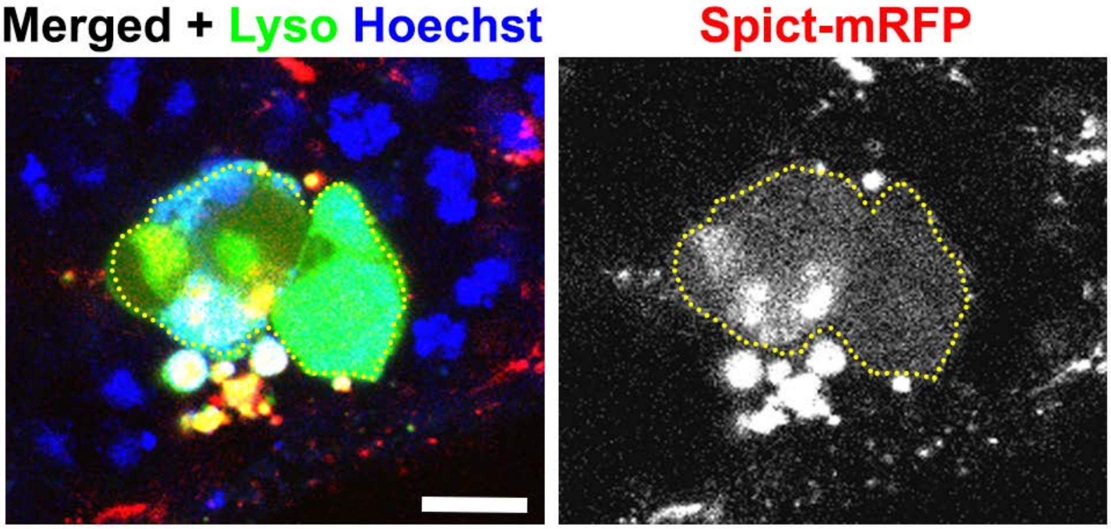

Supplementary Fig. S3. Spict-mRFP expressed by *tj-gal4* is observed in dying SGs.
Expression of Spict-mRFP in CCs using *tj-gal4* results in Spict-mRFP accumulation in dying SGs. N>15testes were examined. Bar: 10μm.

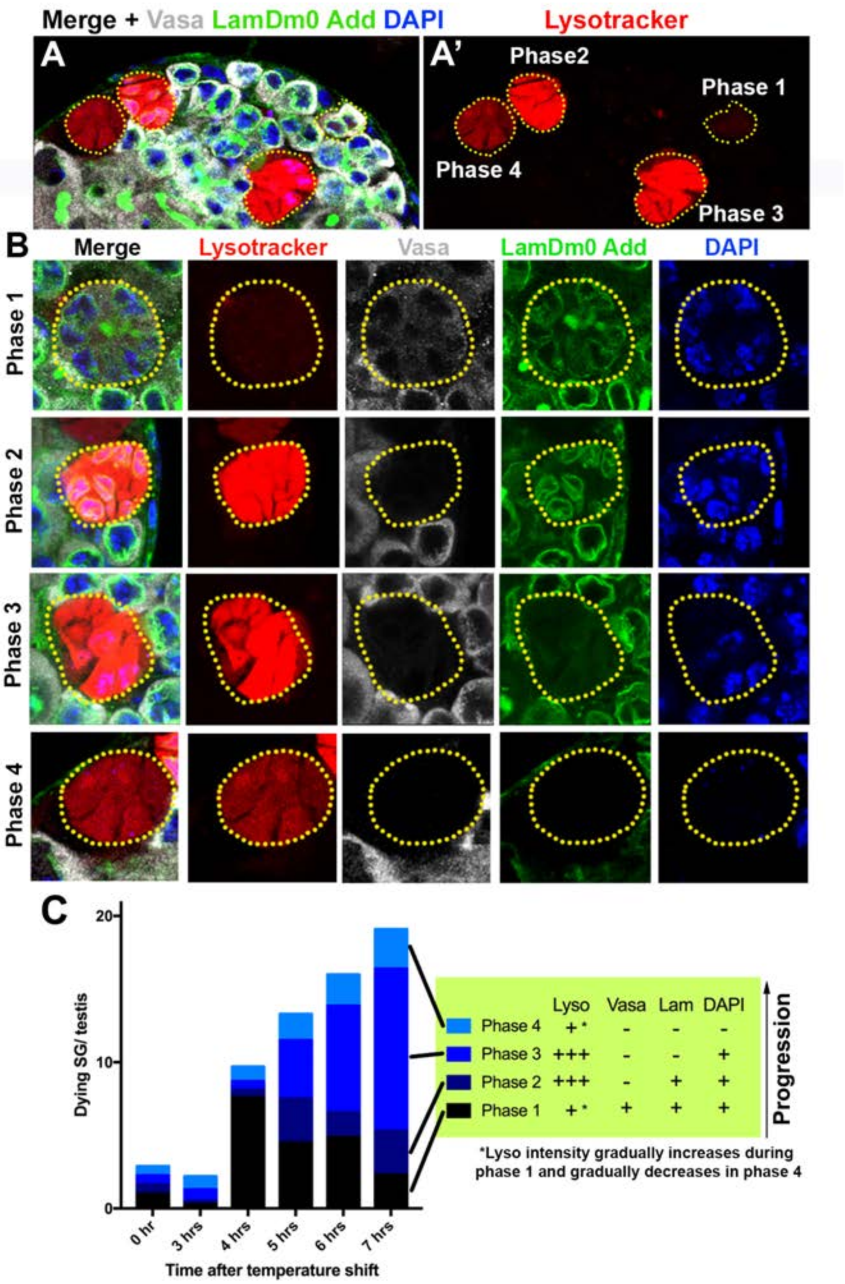

Supplementary Fig. S4. Progression of SG death characterized by Lysotracker, Vasa, LaminDm0, Add and DAPI staining.

(A) An example of a testis apical tip containing multiple phases of SG death.

(B) SG death is divided into 4 phases based on Vasa, LaminDm0, Add, DAPI and Lysotracker staining. In phase 1, SGs becomes weakly Lysotracker-positive, whereas Vasa staining becomes slightly weaker. LaminDm0, Add, and DAPI staining still remains. In phase 2, Lysotracker staining becomes stronger and Vasa staining becomes undetectable, leaving LaminDm0, Add and DAPI staining. In phase 3, Lysotracker staining remains strong, Vasa and LaminDm0 staining become undetectable, DAPI staining remains. In phase 4, all but Lysotracker staining are gone. Lysotracker intensity varies during phases 1 and 4, presumably reflecting gradual increase in Lysotracker intensity during phase 1, and gradual decrease in it during phase 4.

(C) Quantification of SG death phases upon synchronized induction of SG death by expression of Grim in CCs (*tj-gal4; tubP-gal80^{ts}, UAS-Grim*), confirming the order of the 4 phases of SG death. N=7-10 testes were examined for each time point.

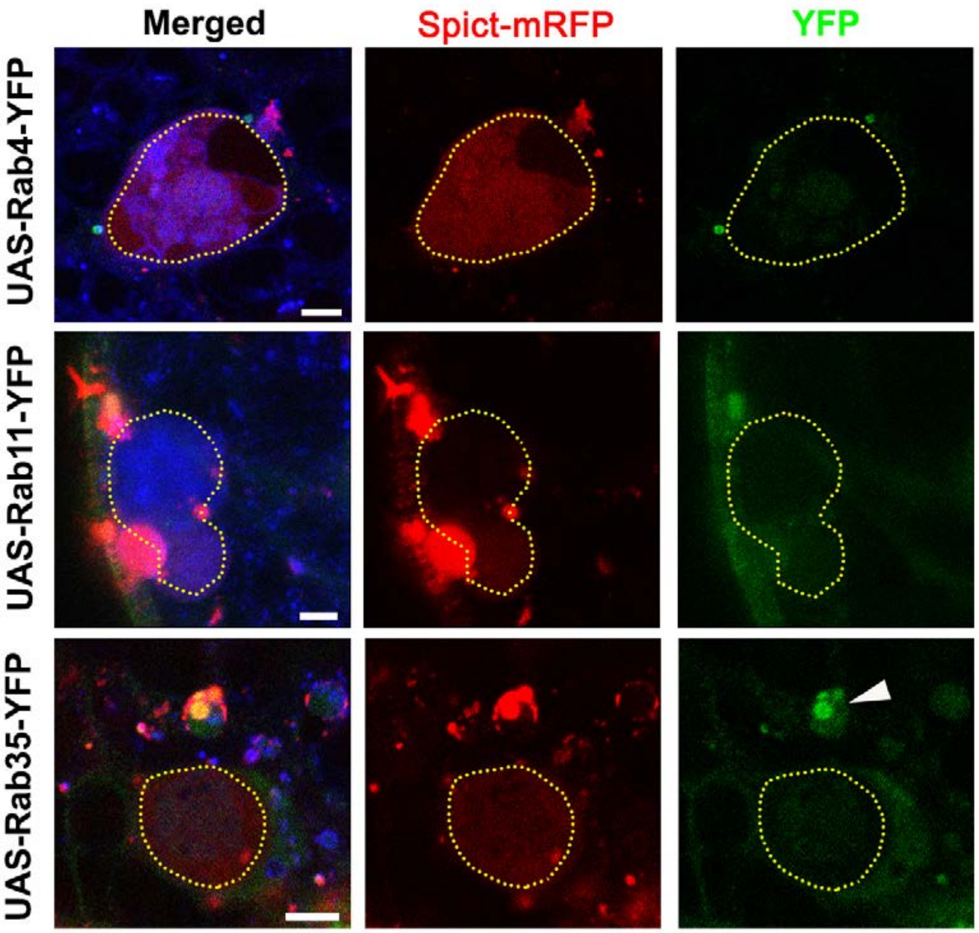

Supplementary Fig. S5. Spict does not colocalize with Rab4, Rab11 or Rab35.
YFP-tagged Rab proteins were expressed under *spict-gal4* driver, together with *UAS-spict-mRFP*. Rab4, 11 or 35 did not show prominent colocalization. Rab35 signal was barely detectable: but when detectable, it was observed to be colocalizing with Spict-mRFP (arrowhead). However, the frequency of visible Rab35 was very low (only one visible punctate signal in every several testes). N>30 testes were examined for each genotype.
